# Sustained and Transient Contributions to the Rat Dark-Adapted Electroretinogram b-Wave

**DOI:** 10.1155/2013/352917

**Published:** 2013-03-06

**Authors:** Trung M. Dang, Algis J. Vingrys, Bang V. Bui

**Affiliations:** Department of Optometry and Vision Sciences, University of Melbourne, 4th Floor, 162 Alice Hoy Building, Monash Road, Parkville, VIC 3010, Australia

## Abstract

The most dominant feature of the electroretinogram, the b-wave, is thought to reflect ON-bipolar cell responses. However, a number of studies suggest that the b-wave is made up of several components. We consider the composition of the rat b-wave by subtracting corneal negative components obtained using intravitreal application of pharmacological agents to remove postreceptoral responses. By analyzing the intensity-response characteristic of the PII across a range of fixed times during and after a light step, we find that the rat isolated PII has 2 components. The first has fast rise and decay characteristics with a low sensitivity to light. GABAc-mediated inhibitory pathways enhance this transient-ON component to manifest increased and deceased sensitivity to light at shorter (<160 ms) and longer times, respectively. The second component has slower temporal characteristics but is more sensitive to light. GABAc-mediated inhibition enhances this sustained-ON component but has little effect on its sensitivity to light. After stimulus offset, both transient and sustained components return to baseline, and a long latency sustained positive component becomes apparent. The light sensitivities of transient-ON and sustained-OFF components are consistent with activity arising from cone ON- and OFF-bipolar cells, whereas the sustained-ON component is likely to arise from rod bipolar cells.

## 1. Introduction

The flash electroretinogram (ERG) provides an excellent noninvasive means to assess retinal function [1]. The largest feature of this response is the b-wave, which follows the initial negative a-wave. The b-wave corresponds to the Granit's [2] PII component and is believed to be generated by the activity of ON-bipolar cells [1]. However, a number of studies have suggested that the PII component may arise from several sources, thus complicating the interpretation of b-wave loss in studies of retinal disease.

Potential contributors to the PII include rod and cone bipolar cell responses, with the later showing faster rise and decay kinetics [3]. ON- and OFF-responses can also be visualized in ERG responses from primates, rabbits, cats, and guinea pigs [4–7]. The responses of ON- and OFF-bipolar cells may have distinct temporal signatures in the b-wave as ON-bipolar cells have been shown to be more sustained compared with their OFF-counterparts in turtle retina [8]. More recent studies suggest that bipolar cells appear to segregate into those producing transient and sustained responses [9, 10]. DeVries et al. [10] have attributed sustained and transient OFF-bipolar cell responses to differences in ionotropic glutamate receptors subtypes: Kainate and AMPA receptors on sustained and transient OFF-bipolar cells, respectively. The temporal characteristics of bipolar cell responses are also thought to be modified by inhibitory processes [11–13]. A number of studies have shown that inhibition of glycine and GABA receptors can modify bipolar cell output [14, 15]. For example, inhibition of GABAc receptors produces more sustained ON bipolar cell responses [16–18] as well as smaller and slower ERG b-wave [17, 19, 20]. More recently, Herrmann and coworkers [21] showed that rod bipolar cell and the dark-adapted b-wave dynamic range in mice are extended by GABAc-mediated sustained chloride current. Whether a similar effect is seen in the rat ERG has yet to be defined.

This paper considers whether the rat PII contains distinct fast and slow subcomponents. It employs several strategies to maximize the possibility for detecting such components. First, the putative PII was isolated by removing photoreceptoral and associated glial and epithelial responses. Second, response amplitudes are considered as a function of stimulus luminance systematically across a range of fixed times. Finally, by applying this analytical approach to data collected following pharmacological manipulation, it is possible to show that the rat ERG b-wave contains distinct fast and slow components. 

## 2. Material and Methods

All animal experimental procedures were conducted with approval from our institutional Animal Experimentation Ethics Committee (A04001) and in accordance with the “Australian Code of Practice for the Care and Use of Animals for Scientific Purposes” [22]. Adult Long Evans rats between 10 and 12 weeks of age (180–270 g, Monash Animal Services, Clayton, VIC, Aust) were maintained at 22°C in a 30–70 lux environment with a 12-hour light/dark cycle (on at 8 am). Water and rat chow were provided *ad libitum*. 

### 2.1. Electroretinography

Animals were dark adapted overnight (>12 hours) and prepared under dim red light (*λ*_max⁡_ ~ 650 nm). Anesthesia was induced with intramuscular injections of 60 mg/kg ketamine (ketamil 100 mg/mL, Troy Laboratories, Smithfield, Australia) and 5 mg/kg xylazine (Xylazil 100 mg/mL, Troy Laboratories). Supplemental injections of half the original dose were given every 50 minutes. Corneal anesthesia was achieved by instillation of one drop of proxymetacaine (Opthetic 0.5%, Allergan, Frenchs Forest, NSW, Australia). Mydriasis was induced with one drop of 0.5% tropicamide (Mydriacyl, Alcon Laboratories, Frenchs Forest, NSW, Australia), giving complete pupil dilation (≥4 mm) within 15 minutes that was maintained for the duration of experiments. Body temperature was maintained (37 ± 0.5°C) by placing the rat over a circulating-water heat pad (MGW Lauda, Lauda-Königshoffen, Germany) and covering the animal with an insulating blanket. The animal and heat pad were mounted on a custom-built platform with the rat's neck and abdomen lightly secured to the stage with Velcro to minimize movement during recordings. 

Full-field ERGs were recorded simultaneously from both eyes using chlorided silver electrodes. The active electrode was placed at the center of the cornea, and the reference was a circular scleral electrode placed around the equator. A stainless steel needle (F-E2-30, Grass-Telefactor, West Warwick, RI, USA) inserted subcutaneously into the tail served as the ground. Following electrode placement 1.0% carboxymethylcellulose sodium (Celluvisc, Allergan, Irvine, CA, USA) was applied to aid electrical conductivity. A readaptation period of 10 minutes in darkness was allowed following electrode placement. The signals were band-pass filtered (0.3 Hz–1 kHz) over a 2560 (640 ms) epoch with a 4 kHz sampling rate. A 50 Hz notch filter was applied *post hoc* to eliminate line noise. 

Stimulus duration of 256 ms was used to better visualize potential slow and fast components. White flashes (5 Watt LEDs, Luxeon, Philips Lumileds Lighting Co., CA, USA) were delivered via a Ganzfeld integrating sphere (total diameter of 36 cm and 13 cm diameter aperture; Photometric Solutions International, Huntingdale, VIC, Australia). Stimulus energy was calibrated using an IL-1700 Research Radiometer (International light, Newbury Port, MA, USA) to return values in scotopic cd·m^−2^. ERG responses were obtained for flash luminances from −2.58 to 2.82 log cd·m^−2^ (−2.58, −2.26, −1.94, −1.54, −0.95, −0.83, −0.62, −0.28, 0.04, 0.36, 0.76, 0.97, 1.27, 1.68, 2.18, 2.53, and 2.82 log cd·m^−2^). The interstimulus interval was increased progressively from 45 to 240 s, to allow for complete recovery of b-wave amplitudes as established in pilot trials.

### 2.2. Intravitreal Injections

As previously described [23, 24], pharmacological agents were injected into the vitreal chamber via a 30-gauge needle attached with polyethylene tubing (inner diameter = 0.38 mm) to a Hamilton syringe (SGE syringes, Ringwood, Australia). The needle was introduced through the pars plana, approximately 1 mm posterior to the superior limbus and at an angle of 45 degrees to avoid the lens. Contact with the lens resulted in opacification, and data from such animals were excluded (<10% of animals). Aliquots of 2–5 *μ*L were used with the upper limit chosen to minimize intraocular pressure changes that might compromise retinal function. Pharmacological agents, 2-amino-4-phosphonobutyric acid (APB, 400 *μ*M), 6-cyano-7-nitroquinoxaline-2,3-dione (CNQX, 250 *μ*M), and (1,2,5,6-tetrahydropyridine-4-yl)methylphosphonic acid (TPMPA, 100 *μ*M) were obtained from Sigma-Aldrich (Sigma-Aldrich Pty. Ltd., Castle Hill, NSW, Australia). APB and TPMPA were diluted using distilled H_2_O, whereas CNQX was mixed in the minimal volume of dimethyl sulphoxide (DMSO) that gave dilution. This DMSO concentration is known to have little effect on retinal function [25]. The concentrations stated represent the estimated final vitreal concentrations assuming complete dilution and a rat vitreal volume of 50 *μ*L [26]. 

A total of thirteen animals received intravitreal injection of the pharmacological agents. Four animals had one eye treated with CNQX, four animals received a combination of APB and CNQX, and the remaining five had one eye treated with TPMPA with the contralateral untreated eye serving as a control in both cases. In addition to the 12 control eyes, an additional ten animals were included as controls in order to increase the size of the normal pool (*n* = 22). Following drug injection, single ERG waveforms were collected at five-minute intervals (stimulus of −0.15 log cd·m^−2^) to track the retinal response for 40 minutes, ensuring a stable drug effect before the ERG protocol began. 

### 2.3. Data Analysis

Average (*n* = 4) APB/CNQX treated signals were subtracted from individual control waveforms at each energy level to remove receptoral, retinal pigment epithelial and glia contributions. Following this subtraction, the ascending limb of the b-wave was described by taking amplitudes at fixed times in 10 ms steps. Amplitudes following stimulus offset were collected at fixed times (every 10 ms) after the waveform had been rezeroed to the amplitude at stimulus offset (256 ms). This step was necessary to remove the influence of the variable decay of the b-wave following its peak.

A hyperbolic function (1) was used to describe the amplitude-energy relationship at various fixed times. The reasoning here is that if the response at a particular fixed time during a light step is mediated by a single underlying generator, then a single hyperbolic function with an exponent of 1 should adequately describe the intensity-response relationship [27, 28]. The presence of a deviation from a single hyperbolic function implies contributions from multiple mechanisms. The function is given by maximal amplitude (*V*_max⁡_, *μV*), semisaturation constant (*K*, log cd·m^2^), and slope (*n*). Consider

(1) $$\begin{array}{c} V\left(I\right)=V_{\max}\cdot \left(\frac{I^{n}}{I^{n}+K^{n}}\right). \end{array}$$

To determine whether data should be fit with a single or multiple hyperbolic function, an *F*-statistic is employed, which provides an unbiased method to assess whether using multiple mechanisms (and additional variables) provides a statistically superior fit (*P* < 0.05) to the data set (e.g., see Figure 4). This approach considers the null hypothesis that a complex model is not a better descriptor of the data than a single hyperbolic function, given the change in the merit function (sum-of-squares) and the degrees of freedom.

### 2.4. Statistics

Statistical analysis was performed using SigmaStat (v3.11, Systat Software Inc., 2004, IL, USA). Two-way repeated measures (RM-)ANOVA was conducted to consider interactions between treatment and stimulus energy. A Holm-Sidak Post Hoc test was applied following two-way RM-ANOVA to consider the source of significant differences (*P* < 0.01). The Holm-Sidak protects against increased Type 1 errors associated with multiple comparisons but is considered more powerful than the Bonferroni correction [29]. Throughout this study, group data are reported as means ± SEM. 

## 3. Results

**Figure 1:**
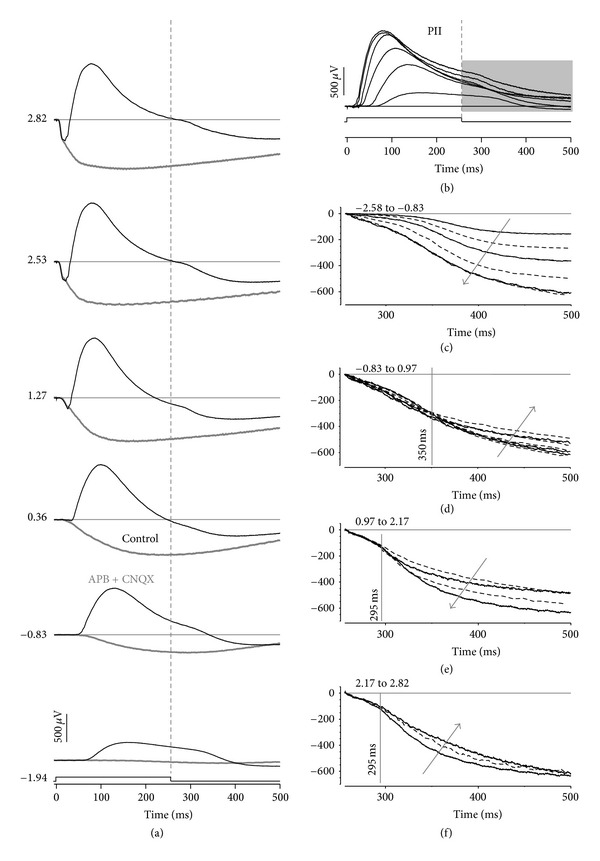
Rat electroretinogram in response to a light step. (a) Average waveforms for animals treated with APB/CNQX (thin grey) compared to untreated control eyes (black). The numbers along the right hand side represent the luminance in log cd·m^−2^. (b) The responses from photoreceptoral, photoreceptoral-driven glial, and RPE responses are removed by subtracting APB- and CNQX-treated waveforms. (c)–(f) Response following light offset for control data. The waveform for the brightest luminance in each panel is repeated in the following panel to aid with comparisons. The grey arrows indicate growth in amplitude with increasing luminance. (c) and (e) show a growth in amplitude (i.e., more negative values), whereas (d) and (f) show a reduction in amplitudes. The vertical lines indicate approximate times up to which the waveforms can be seen to share a common shape. The luminance quoted in the panel labels is in log cd·m^−2^.

Figure 1 shows representative control and drug-treated waveforms in response to 256 ms flashes. Figure 1(a) shows that at dimmer luminances, the ERG waveform is dominated by a corneal positive b-wave. The response remains sustained for 100 ms following light offset before it shows relaxation back to baseline. The relaxation overshoots the initial baseline to be corneal negative at 150 ms after flash offset. At higher luminances, both the a-wave and b-wave become more apparent. The b-wave develops an early transient peak (100 ms) followed by a slower tail and finally amplitude relaxation, after stimulus offset. 

Following application of APB/CNQX (thin grey line), the b-wave is completely abolished, leaving a corneal negative waveform that grows in amplitude with higher light levels. Figure 1(b) shows the putative PII following subtraction of APB/CNQX-treated signals to remove presumed contributions from photoreceptors, RPE, and glial cells (thin grey line of Figure 1(a)). This approach shows that the putative PII is sustained at low light levels and becomes more transient at brighter levels. 

Figures 1(c) to 1(f) consider the response following stimulus offset after signals are rezeroed to the amplitude at 256 ms (stimulus offset). The amplitude and timing characteristics of the post-offset relaxation have several phases. At low light levels, from −2.58 to −0.83 log cd·m^−2^, increasing luminance results in a progressively larger (more negative) relaxation (Figure 1(c), direction of grey arrow). For stimuli between −0.83 and 0.97 log cd·m^−2^, however, the relaxation becomes progressively smaller (Figure 1(d), grey arrow), but only after a latency of 100 ms after stimulus offset. Between 0.97 and 2.17 log cd·m^−2^, the relaxation at stimulus offset again becomes more negative, but only after a latency of 40 ms (Figure 1(e)). For brighter light levels of 2.17 to 2.82 log cd·m^−2^, the relaxation amplitude tends to become smaller again (Figure 1(f)). 

### 3.1. Modulation of Ionotropic Neurotransmission

**Figure 2:**
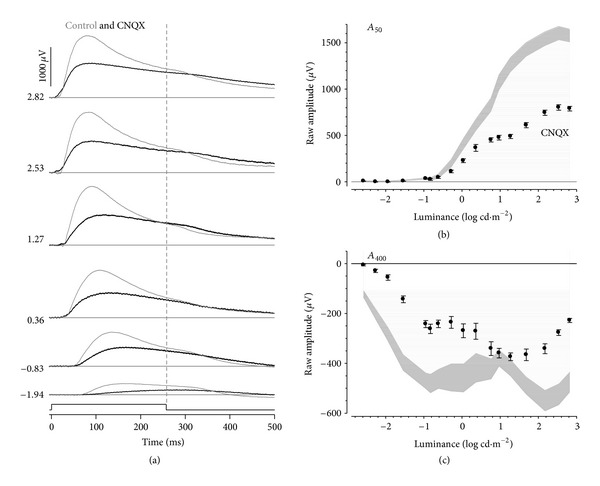
Effect of CNQX on rat-isolated PII. (a) Representative CNQX waveforms (thick) compared to its fellow control eye (thin). Luminance is given in log cd·m^−2^ along the left. Grouped average (±SEM) amplitudes at fixed times of 50 (b) and 400 ms (c) are plotted against luminance. CNQX-treated (black filled) data are compared against control eyes (grey areas indicate 95% confidence intervals of controls as calculated from the SEM).

Figure 2(a) overlays CNQX (black line) and control (thin grey line) PII waveforms and shows that application of CNQX reduces the early transient peak of the b-wave. In addition, the relaxation after stimulus offset appears delayed with CNQX. Figure 2(b) compares control (grey area represents the standard error of the mean) and CNQX-treated amplitudes at a fixed time of 50 ms (*A*_50_). CNQX intensity-response functions show a plateau between 1.27–1.68 log cd·m^−2^ and a secondary plateau at higher luminances, suggesting that at least two mechanisms contribute to the isolated PII under these conditions. Two-way ANOVA reveals significant reductions in amplitudes (*P* < 0.035; treatment *x* luminance interaction, *F*_1,17_ = 59.8, *P* < 0.001) for stimulus luminances greater than 0.04 log cd·m^−2^.

Figure 2(c) shows that at *A*_400_ (144 ms after offset), the control intensity response-function shows an initial growth in amplitude that makes a plateau at approximately −0.95 log cd·m^−2^. This is followed by a second phase of amplitude growth (between 1 and 2 log cd·m^−2^) and finally a reduction in magnitude at the highest luminous energies (>2 log cd·m^−2^). CNQX application resulted in a second phase of amplitude growth that was approximately 1 log unit more sensitive compared with the control data. 

### 3.2. Modulation of GABAergic Neurotransmission

**Figure 3:**
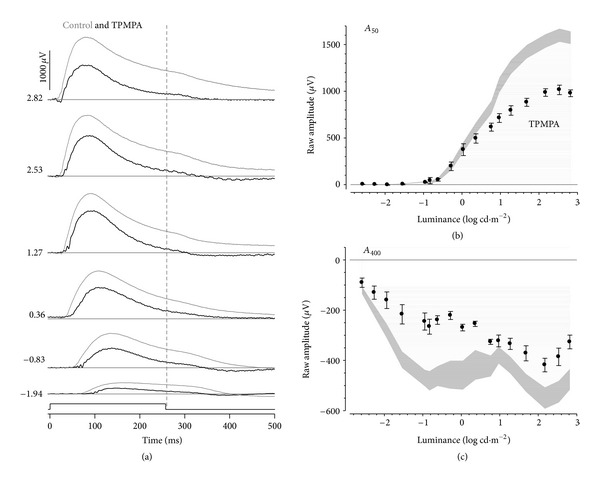
Effect of TPMPA on rat-isolated PII. (a) Representative TPMPA waveforms (thick) compared to its fellow control eye (thin). Luminance is given in log cd·m^−2^ along the left. Grouped average (±SEM) amplitudes at fixed times of 50 (b) and 400 ms (c) are plotted against luminance. TPMPA-treated (black filled) data are compared against control eyes (grey areas indicate 95% confidence intervals of controls as calculated from the SEM).

Figure 3(a) shows representative PII waveforms following inhibition of GABAc receptors (black line) compared with controls (thin grey line). TPMPA treatment resulted in smaller b-wave amplitudes particularly for brighter luminance. The offset relaxation was less apparent following TPMPA treatment, consistent with smaller onset amplitudes. Analysis of the intensity-response characteristics at *A*_50_ (during light step, Figure 3(b)) and *A*_400_ (after light step, Figure 3(c)) shows that TPMPA resulted in changes that are qualitatively similar to CNQX treatment (see Figure 2). Specifically, at *A*_400_, TPMPA resulted in reduced amplitudes as well as a more sensitive second phase of amplitude increase. 

### 3.3. Modeling Contributions to the PII during a Light Step

**Figure 4:**
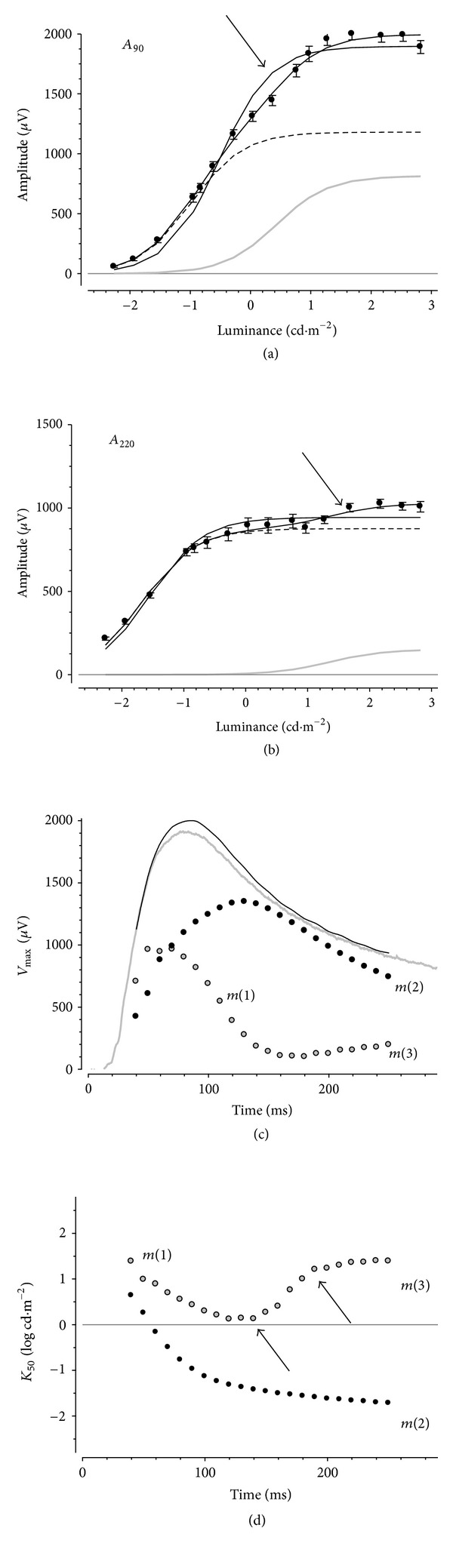
Modeling contributions to rat PII during a light step. Average amplitudes (±SEM) as a function of luminance at different fixed times (90 and 220 ms) after stimulus onset. (a) and (b) show a single hyperbolic function fit to the data (thick grey line). Arrows indicate the possible presence of a secondary mechanism at brighter intensities. A combination of two hyperbolic functions (grey trace and dashed trace) provides a better fit to the data than a single hyperbolic function. The black trace shows the sum of the two hyperbolic functions. The optimized *V*_max⁡_ (c) and *K*_50_ (d) parameters are plotted as a function of time for the two hyperbolic functions. (c) shows the combined amplitude for the two hyperbolic functions (black trace) compared with the actual waveform for the 2.82 log cd·m^−2^ (grey trace) stimulus. The arrows in (d) indicate changes in *K*_50_, which are likely to represent a transition between mechanisms.

Figures 4(a) and 4(b) show average amplitudes at selected fixed times optimized with a hyperbolic function (representative fixed times of *A*_90_ and *A*_220_). At these fixed times, a simple hyperbolic function (thick line) fails to fully describe the data, suggesting the presence of more than one component. The arrows show the transition between the two intensity-response phases. The same data are better modeled by the summation (thin solid line) of two hyperbolic functions (dashed and grey lines), both having their exponents constrained to one. An *F*-test showed that a significantly better fit to the data could be achieved with the two hyperbolic functions (*P* < 0.05). 

Figures 4(c) and 4(d) show optimized *V*_max⁡_ and *K*_50_ plotted as a function of fixed time for the two components, which have been designated as *m*(*1*) and *m*(*2*). The parameters show a systematic change across time. In Figure 4(d), it was apparent that the two hyperbolic functions show distinct sensitivities (*K*_50_). If at each time we designate those hyperbolic functions that show larger *K*_50_ as *m*(*1*) and those with smaller *K*_50_ as *m*(*2*), then the corresponding *V*_max⁡_ values for *m*(*1*) and *m*(*2*) are shown in Figure 4(c). This separates the two components into a transient and a sustained process. Figure 4(d) shows that the less sensitive transient *m*(*1*) component underlies the earliest part of the isolated PII. Beyond a fixed time of 80 ms, the more sensitive but sustained *m*(*2*) comes to dominate the rat PII. Interestingly, at later fixed times (>170 ms), another feature appears to grow in amplitude to contribute ~20% to the PII. This secondary amplitude growth, designated as *m*(*3*), may reflect the presence of an additional slower component or the relaxation of *m*(*1*) to a baseline during a light step. The *K*_50_ for *m*(*1*) (Figure 4(d)) shows a transition at 170 ms, to become less sensitive (arrows) consistent with the possibility of intrusion from another component. Its intensity-response function reveals a low sensitivity, suggesting the possibility that it might arise from the cone pathway. The waveform designated *m*(*2*) shows an increase in sensitivity to plateau at later times, consistent with a single component.

**Figure 5:**
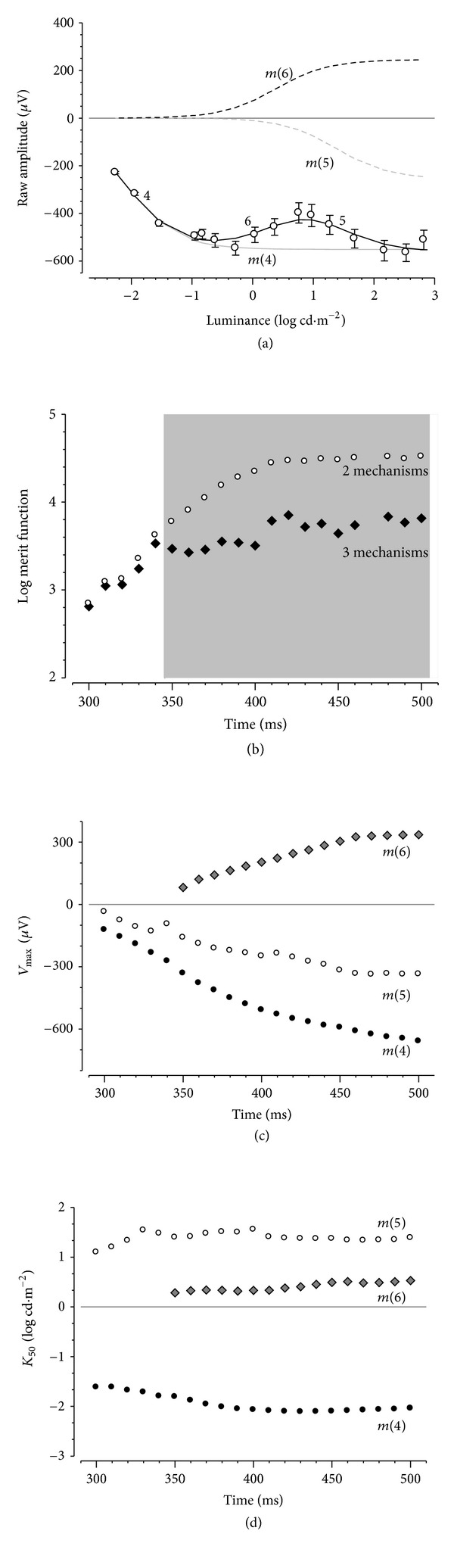
Modeling contributions to rat PII after a light step. Average (±SEM) amplitudes following stimulus offset at a fixed time of 400 ms are modeled with three mechanisms. The two negative components (4 and 5) are modeled with two negative hyperbolic functions (*m*(*4*) solid grey line and *m*(*5*) dashes grey line), whereas the positive portion (6) is fit with a positive mechanism (*m*(*6*) black dashed line). The solid black line represents the summation of the three hyperbolic functions fit to the raw data. (b) The merit functions show that three hyperbolic functions (filled) provide statistically better fit for fixed times >340 ms (grey area, *P* < 0.05) compared with two hyperbolic functions (unfilled). *V*_max⁡_ (c) and *K*_50_ (d) plotted as a function of fixed times.

Figure 5(a) applies the same modeling approach to amplitudes at various fixed times following stimulus offset. The example shown here is for the fixed time of *A*_400_, which shows an initial growth in amplitude, followed by a reduction in magnitude between −0.62 and 1.27 log cd·m^−2^ and finally a second phase of amplitude growth. This intensity-response profile cannot be modelled using one or even two hyperbolic functions. Indeed, it can only be adequately modeled with three functions (Figure 5(a)), the two negative components designated *m*(*4*) and *m*(*5*), with the addition of a positive component designated *m*(*6*). 

The merit function (log sum of square (SS) error) for the two- and three-component models was compared using an *F*-ratio. Figure 5(b) shows that beyond a fixed time of 340 ms, the addition of a third positive hyperbolic function provides a statistically smaller SS compared with a two-function model. The *V*_max⁡_ parameters for the three components increase with longer fixed times as shown in Figure 5(c). Figure 5(d) shows that the *K*_50_ parameters for the three components are distinct from each other and remain relatively constant across the range of fixed times after a light step. 

**Figure 6:**
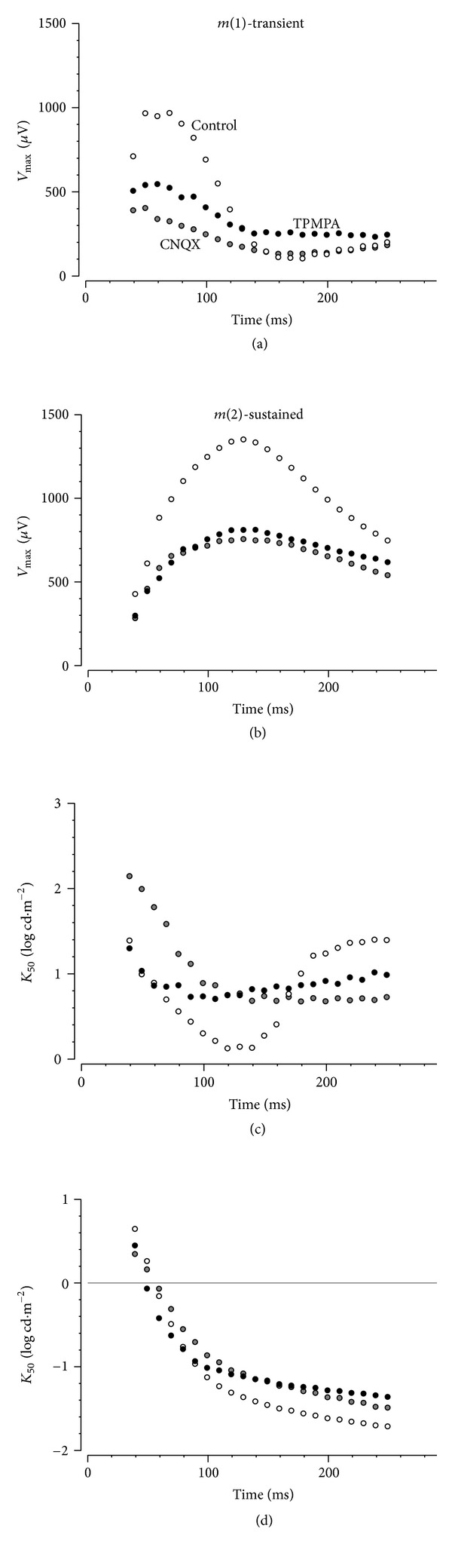
Effect of CNQX or TPMPA on rat PII during a light step. Optimized parameters *V*_max⁡_ ((a) and (b)) and *K*_50_ ((c) and (d)) for controls (unfilled symbols), CNQX (grey), and TPMPA treated (black symbols). Left panels show the transient mechanism (*m*(*1*)), whereas the right panels show the sustained mechanism (*m*(*2*)).

Figure 6 shows the effect that CNQX and TPMPA have on the parameters of the putative *m*(*1*) and *m*(*2*) components. For *V*_max⁡_, *m*(*1*) was reduced by CNQX treatment (~59%) at early fixed times (<150 ms), whereas *m*(*3*) appeared to be unaffected. TPMPA produced a smaller attenuation of *m*(*1*) (~44%, Figure 8(a)) but produced a small enhancement of *m*(*3*). In contrast, Figure 6(b) shows that both CNQX and TPMPA produced similar effects on the sustained component (*m*(*2*)).

Figure 6(c) shows that application of CNQX removed the transition in *K*_50_ between the *m*(*1*) to *m*(*3*) components. Likewise, TPMPA application also removed this transition; however, *K*_50_ was larger for early times with TPMPA. This pattern of *K*_50_ change following CNQX or TPMPA suggests that *m*(*1*) may be a common process whose sensitivity is differentially modified at early (more sensitive, <150 ms) and later fixed times (less sensitive, >180 ms). Figure 6(d) shows that CNQX and TPMPA had little effect on the *K*_50_ of *m*(*2*). 

### 3.4. Modeling Contributions to the Rat PII after a Light Step

As was the case for the control data, those recorded following CNQX and TPMPA application were evaluated to consider if three components are evident following a light step. In both CNQX- and TPMPA-treated data, the log merit function shows that there was not a significant reduction in error to warrant the use of the more complex three hyperbolic function models; thus, only two are needed to explain the trends in the drug-treated eyes. This suggests that the positive going *m*(*6*) was eliminated by the drug treatments.

**Figure 7:**
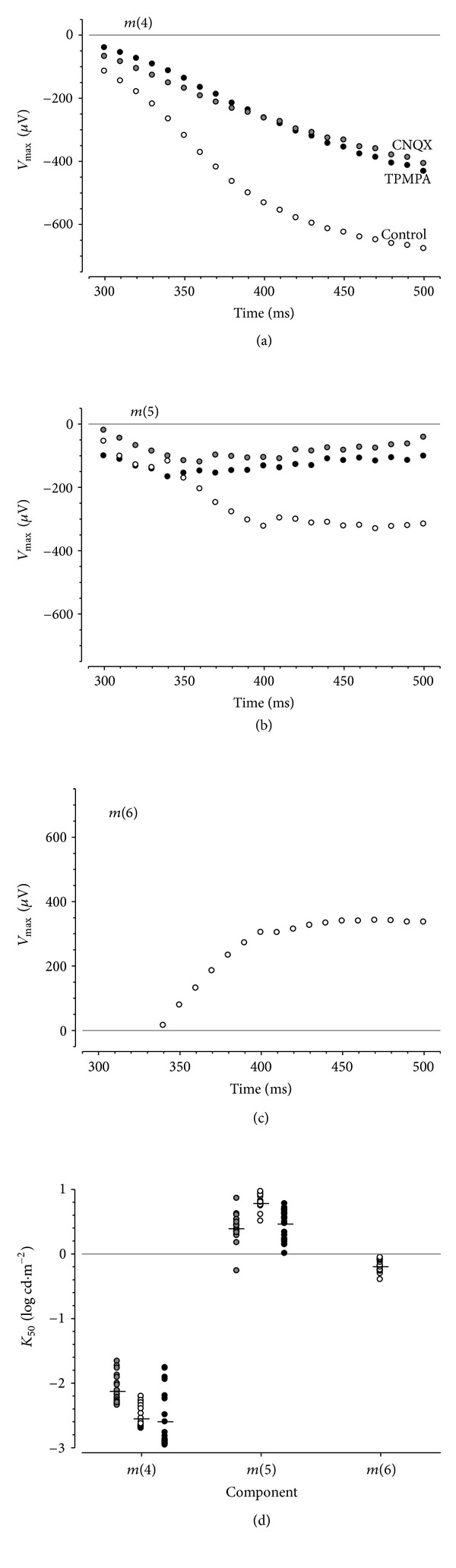
Effect of CNQX or TPMPA on rat PII after a light step. *V*_max⁡_ is shown for *m*(*4*) (a), *m*(*5*) (b), and *m*(*6*) (c) mechanisms as a function of fixed time from light offset for controls (unfilled symbols), CNQX (grey symbols), and TPMPA (black symbols). (d) shows *K*_50_ for the three mechanisms for controls (unfilled), CNQX (grey), or TPMPA treated (black). The average for each group is indicated.

Figures 7(a) and 7(b) show *V*_max⁡_ after a light step derived from control and drug-treated data for *m*(*4*) and *m*(*5*). Eyes treated with CNQX or TPMA returned smaller amplitudes compared with controls. The reductions in amplitudes after a light step may simply reflect drug-induced reductions in amplitudes during the light step. The *K*_50_ is shown for each function in Figure 7(d). A *t*-test shows for *m*(*4*) that the CNQX *K*_50_ is larger than that of controls (control: −2.55 ± 0.04, CNQX: −2.12 ± 0.05; *P* < 0.001), whereas TPMPA was similar. For *m*(*5*), both CNQX and TPMPA treatments produced *K*_50_'s that were smaller than controls (control: 0.78 ± 0.02, CNQX: 0.39 ± 0.04, TPMPA: 0.47 ± 0.05; *P* < 0.001).

## 4. Discussion

We find that the isolated PII (control waveform—APB/CNQX sensitive waveforms) in the pigmented rat electroretinogram has complex luminance response characteristics during and after a 256 ms light step. Our approach of modeling the intensity-response function across a range of fixed times revealed that during a light step, the rat PII contains transient and sustained responses, which relax to baseline after the light step. In addition, there was a slow corneal positive response that follows stimulus offset. 

### 4.1. Contributions to the Rat PII during a Light Step

Consistent with previous studies of rodent b-waves [28, 30], we also find that the isolated PII amplitude (in our case for a fixed time of 50 ms, *A*_50_) intensity-response function returned a slope of 0.99 (0.89, 1.09) (2.5%, 97.5%). Thus, a single hyperbolic function can describe the earliest portions of the rat PII leading edge. However, a single hyperbolic function cannot fully describe the intensity-response data for later fixed times after stimulus onset. Such complex behavior has previously been attributed to interactions between negative PIII and positive PII components [31–33]. However, even after subtracting corneal negative waveforms isolated using APB/CNQX, the PII intensity-response function retains its complex profile, suggesting that there are multiple components underlying the response.

To model the amplitude, intensity-response function extracted at fixed times after stimulus onset required two hyperbolic functions (Figure 4), which segregate into processes that have high and low sensitivities to light (Figure 4(d)) and are sustained (*m*(*2*)) and transient (*m*(*1*)) (Figure 4(c)), respectively. This is consistent with previous studies suggesting that the b-wave contains sustained and transient responses (also termed slow and fast PII) [1, 9, 34, 35]. Here, we show that these sustained and transient components do not arise from interactions with corneal negative responses. Application of CNQX to block ionotropic glutamate receptors does not completely remove either *m*(*1*)* or m*(*2*), indicating that the transient and sustained responses do not arise simply from ON- and OFF-bipolar. 

In addition to these sustained and transient components, there appears to be a third longer latency (140–190 ms) component (designated as *m*(*3*)). This component has low amplitude (~ 20%, Figure 4(c)) and is less sensitive than *m*(*1*) for times longer than ~140 ms (Figure 4(d)). Treatment with either CNQX or TPMPA removes the transition of *K*_50_ from a more sensitive *m*(*1*) to a less sensitive *m*(*3*), producing a *K*_50_ profile that resembles a single mechanism (Figure 6(c)). This outcome suggests that *m*(*3*) is likely to be a continuation of *m*(*1*) during a light step.

Lateral elements in the IPL, in particular amacrine cells, can modulate the ON-bipolar cell response and the ERG b-wave [11, 17, 19, 36, 37]. Inhibitory input has been shown to confer transient and sustained characteristics to bipolar cell neurotransmitter release [11, 38]. Recordings from single cells suggest that such inhibition occurs via GABAa and GABAc receptors onto rod and cone ON-bipolar cells in rats [39]. Eggers et al. [40] show that higher proportions of GABAc to GABAa receptors-mediated inputs produce more prolonged inhibition, whereas a relative higher proportion of GABAa to GABAc results in more transient inhibition. This is consistent with the finding that inhibition of GABAc receptors using TPMPA makes bipolar cell responses in mouse, rat, and amphibian retina more sustained [16, 39] with a smaller response range [21]. A less hyperpolarized resting membrane potential arising from loss of a tonic GABA-mediated current [21, 41] would account for smaller and more sustained b-wave. Herrmann et al. [21] provide compelling evidence that dopamine-mediated GABA release possibly from horizontal cells, acting on both GABAa and GABAc receptors, modulates the dynamic range of rod bipolar cells via a sustained Cl^−^ current.

Activation of GABAc receptors increases presynaptic Cl^−^ influx, hyperpolarizing bipolar cell and thereby inhibiting Ca^2+^ entry via L-type Ca^2+^-channels [11, 21, 38]. Studies have shown that L-type Ca^2+^ channels may be differentially modified as a function of time following light onset [38, 42], implying that the ON-bipolar cell responses may be both enhanced and inhibited at different times. In rat retina, A17 amacrine cells are known to make reciprocal synapses onto rod bipolar cells [11, 43]. It has been suggested, based on data from tiger salamander retina, that a glycinergic amacrine cell initially inhibits the A17 amacrine [42], thereby delaying its action at GABAc receptors on ON-bipolar cell axon terminals. In this way, amacrine cell inhibition of bipolar cells will occur only after the glycinergic amacrine cell inhibition onto the A17 amacrine cell is removed (estimated to be ~150 ms, [42]). This mechanism provides an explanation for our finding that GABAc-mediated inhibition produces a more transient PII component, by differentially modifying the PII at early and later times. Specifically, our TPMPA data shows that there is amplitude enhancement and increased light sensitivity from 70 to 160 ms after onset of the light step, as well as a decrease in light sensitivity for times later than 180 ms (Figure 6(c)). Moreover, that CNQX results in the same qualitative outcome is consistent with amacrine cell involvement, as AMPA/KA inhibition will affect both glycinergic and GABAergic amacrine cells. Herrmann et al. [21] suggest that dopamine released from amacrine cells might also modulate the tonic GABA currents at the level of rod bipolar cell dendrites. Thus, modification of bipolar cell responses might occur at both outer and inner plexiform layers [21].

It is possible that transient and sustained components described here can reflect both cone and rod bipolar cell responses. The difference in response range of *m*(*2*) and *m*(*1*) might suggest rod and cone ON-bipolar cell contributions to these components, respectively. Previous work by Nixon and colleagues [44] has shown that the rod b-wave is ~1.5 log units more sensitive than the cone b-wave for short duration flashes. This difference is comparable to that found at early fixed times for *m*(*1*) and *m*(*2*) seen here (<120 ms, ~1.5 log units). However, interpreting *m*(*1*) and *m*(*2*) to be cone and rod mediated needs to guarded as responses from single rat rod bipolar cells to a step of light have a faster onset [27] than the *m*(2) components isolated in this study.

### 4.2. Contributions to the Rat PII following Stimulus Offset

The response following the offset of a light step could not be modeled with two components and required a third hyperbolic function (Figure 7). At very dim intensities, only one component appeared to contribute to the signal (*m*(*4*)). However, with brighter intensities, two additional components become apparent, which are opposite in polarity (*m*(*5*) negative and *m*(*6*) positive). These offset components are a relaxation of *m*(*2*) and *m*(*3*) back to baseline. Indeed, the *K*_50_ values for *m*(*4*) and *m*(*5*) correlate well with those for *m*(*3*) and *m*(*2*) (compare Figure 7(d) to 6(c) and Figure 7(d) to 6(d), respectively, consistent with this proposal. This possibility is also supported by the findings that CNQX (reduction at *A*_50_50.7 ± 1.5% and *A*_400_ 56.1 ± 4.1%, *P* = 0.19) and TPMPA (reduction at *A*_50_ 41.9 ± 2.5% and *A*_400_ 41.1 ± 5.0%, *P* = 0.40) induce comparable reductions in amplitude for both onset (*A*_50_) and negative offset components (*A*_400_).

A corneal positive component (Figure 7(c)) apparent following stimulus offset (here designated *m*(*6*)) was only present in control eyes. Naarendorp and Williams [45] have shown that positive OFF-component arising after a light step in rat was abolished after inhibition of ionotropic glutamate receptors, indicating an origin from hyperpolarizing bipolar cells. Our finding that the *m*(*6*) was removed by CNQX, which inhibits ionotropic receptors, correlates well with this interpretation. However, TPMPA also altered this positive OFF-component. Hence, this component may also be modulated by disruption to inner retinal feedback as it is known that CNQX also modifies amacrine cell function. 

## 5. Summary

**Figure 8:**
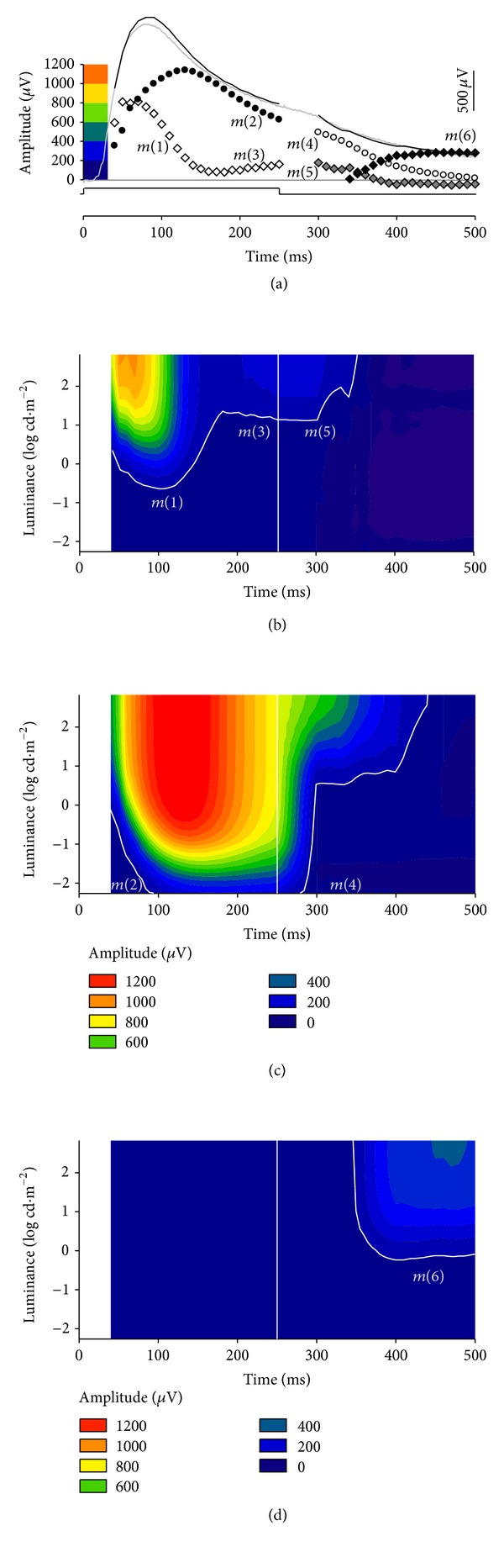
Summary of components underlying the rat PII response to a light step. (a) The interactions between the various components derived from our modeling are shown for the brightest luminance. Hence, there appear to be a minimum of three components underlying the scotopic ERG PII elicited using a light step. (*m*(*1*) and *m*(*3*): unfilled diamonds, *m*(*2*): black circles, *m*(*4*): unfilled circles, *m*(*5*): grey diamonds, and *m*(*6*): black diamonds). (b)–(d) show individual components plotted on a contour plot, as a function of time and luminance, with larger amplitudes indicated by warmer colors. (b) *m*(*1*) has fast rise and decay characteristics with a low sensitivity to light. With longer times *m*(*1*) transitions to *m*(*3*). After stimulus offset, *m*(*3*) continues as *m*(*5*) and relaxes back to baseline. (c) *m*(*2*) has slow rise and decay characteristics and is more sensitive to light. After stimulus offset, *m*(*2*) continues as *m*(*4*) and relaxes back to baseline. (d) A long latency small amplitude sustained positive component *m*(*6*) occurs after stimulus offset at higher luminances.

Putative components *m*(*1*) to *m*(*6*) are plotted in Figure 8 to yield the composite PII waveform in response to a 256 ms light step. Based on our analysis, we believe that the isolated scotopic PII in rats contains at least three separate components. During a light step, there is an initial transient-ON response, which is made more transient by GABAc-mediated inhibitory processes. The majority of the PII for times greater than ~100 ms reflects a sustained-ON component, likely arising from rod bipolar cells. After a light step, transient-ON and sustained-ON components relax to baseline. In addition, there is a positive sustained-OFF component that is likely to arise from the OFF-pathway, but it may also be modulated by GABAc receptors. Thus, assessing the intensity-response functions at early and later times during a light step provides a means by which transient and sustained contributions to the rat PII ERG can be identified.
